# Supplementary material for: An invisible caregiver for visibly older parents: Experiences of (young) adults shared as comments to newspaper articles on advanced age parenthood
Source: PLoS One. 2023 Nov 30;18(11):e0295018. doi: 10.1371/journal.pone.0295018 (PMC10688717; doi:10.1371/journal.pone.0295018)
Supplement: S1 Dataset — (PDF) [file pone.0295018.s001.pdf]

Supporting information: Dataset

Comments from (adult) children born to older parents as shared to newspaper articles in The Guardian and The Daily Mail Online

All usernames or real-life names associated to the collected comments have been replaced by 'commenter' followed by a digit.

Some comments were formulated as a reply to another comment. In the cases in which the latter comment was written by a commenter that we did not include in this dataset (because this commenter did not identify as a child born to older parents) we replaced their username or real-life name to 'external commenter'.

Comments to newspaper articles in The Guardian

Commenter 1
My dad was 49 when I saw born. He used to say that my sister and I kept him young. I never really noticed his age. He was a great dad.

Commenter 2
When I was born my Dad was 50. He once apologised for not being more active when I was a kid. I told him not to be daft and that I had a great childhood. I did and I don't feel that I missed out. Dad is no longer with us. He died 17 years ago. I am 47. I miss being able to share things with him in my middle age. I have always missed our chats. It will be the centenary of his birth in 2019. I plan to do something special to mark it. I live in Sydney now but I have one special day when I always stop and do something for my Dad: Armistice Day 11/11. He fought in WWII, never talked about the horrors he saw but we always went to remember his mates together. So this is one way I stay connected to him. Love and miss him a lot.

Commenter 3
A lovely article thank you. My dad was 44 when I was born, my mum 30. I had a truly lovely, generous, active, supportive dad and I realised long ago that youth doesn't make you a more active parent, good health does. I recognise so many of the stories, the surprise of your school mates, the wonder of my class at his gas mask from being a child of the war, and my entire GSCE year benefitting from his cracking of the maths coursework. grief is the price you pay for love....how true and how worth it.

Commenter 4
My dad was fifty when I was born and died, holding my hand, when he was 93. He had fought in WW2, and then, having began a new career in teaching, enabling the next generations to make the most of the freedoms he had fought to preserve. He was intellectual, politically passionate, notoriously frugal and fairly eccentric. I feel his influences every day, and my only regret at having an older dad is that my own kids didn't get to know him in his prime. I see a lot of him in my teenage daughter, and yesterday, as she was ranting about the outcome of the election, I glanced at his framed photo and could have sworn I caught amusement in his eyes...

Commenter 5
A beautiful article. My father was in his 50's when I was born and as a child I used to worry all the time about him dying. He's 94 now, physically frail but mentally and emotionally

wonderful. He never played with me but he talked to me and listened to me with calm and grace. I am grateful for having my dad as a dad.

Commenter 6

What a nice article. I can, to a degree, understand what some of the interviewees went through, though my father was a 'mere' 43 when I was born (Mum was 37). I was glad as a teenager that he always looked about 10 years younger than he actually was but stopped caring about it, as I got older.

I love my Dad, he's one of the nicest people I've ever met and I feel immensely proud and lucky that I've had him as a parent. It's sad seeing him become frailer, and I worry that if I don't find someone to settle down and have kids soon, he may never see my children (assuming I ever have them). But when I feel sad, or worry about that, I try to remember how lucky I've been to have him as my Dad; how many people lose parents who are younger than my father, or who have abusive parents. I wouldn't change him for anyone else.

Commenter 7

Commenter 6

Your parents were about the same age as mine when i was born. I was always aware that they were much older than friends parents growing up. They didn't have the same interests or attitudes and i always worried about them getting frail and old - my mum died a couple of years ago now and my dad has mid stage dementia and it has all happened at an age when i'm still trying to sort my own life out and it's been very hard to cope with. That is why now i am 39 and not married I think i am too old to have children now as i wouldn't want to put the same burdens on them as I have had.

Commenter 8

My Dad served in WW2 and then did his law degree. He was in his late 30's before he was able to marry and start a family. He was already 40 when I was born (I have an older brother and younger sister). We three children always felt that our parents were from another generation entirely, preferring Frank Sinatra to the Beatles and fiercely rejecting the cultural changes of the 60s & 70s. Dad worked incredibly long hours, conscious of how few earning years he had to provide for the 3 of us, and then his own pension, and Mom was increasingly incapacitated by arthritis, which began in her 30s. So we couldn't physically play with our parents, and fought ferocious cultural & political battles with them as teens. As a result, both my siblings chose to start families when they were in their 20s, and now, as we all reach middle age, they are enjoying having grandchildren whilst still being relatively young & fit themselves. Whereas we had to deal with our parents' health problems whilst still children ourselves, my nieces & nephews were able to rough-house & play with their parents uninhibitedly and now their children have active, healthy grandparents. I'm very proud of what my father achieved in his lifetime, but at one point, everyone in the family was struggling with looking after ancient grandparents (in their 90s), elderly parents (in their 70s) and small babies. I'm not sure children should have the burden (and worry) of caring for elderly parents when they're still growing up themselves. People say "Oh they keep me young." Isn't that narcissistic? It's not their job to keep you anything - it's the parents' job to raise the child, not the child's job to shoulder responsibility for the parent.

Commenter 7

External commenter

As a child of older parents i think Emmi26 is referring to the cultural gap between her parents and her peers' parents rather than between parents and children. My parents had no interest in

socializing with my friends parents because they were from different eras with different interests.
I also recognise the wish to have a family at a young age as a reaction to this - i desperately wanted to get married from the age of 19, but have reached 39 without having achieved this and now i will probably never get married and definitely never have children. And f course, i also have had the responsibility of sick and dying parents at an age when my peers are having holidays and theatre trips with their parents.

Commenter 9

dtr1001 (Isn't that just selfish? Of course it is the child's responsibility to care for the parent as they become infirm. What a bizarre thing to say.)

Or maybe it is selfish to bring a child into the world in older age and then expect them to care for you ...

Commenter 10

my dad was 56 when i was born, and died last year aged 91. He was a bomber pilot at the end of the war, something of which I never ceased to be immensely proud. But more than that he was the person I had fun with, who taught me to ride a bike, who cycled by my side through country lanes, hid forbidden chewing gum, took me to new places, and shared everything with me. I always worried that he wouldn't be there for long enough, but maybe that makes each moment more precious - and as luck would have it my much younger mother (21 years his junior) died young so it was my older dad who was there for longest. In recent years we revisited places from my childhood together with my son and I cannot describe the joy that this brought to all three of us.

Commenter 9

External commenter

Yes. This is a common theme in discussions. Where the female part of the couple does not have health issues / die then she becomes the primary carer. Her choice. Maybe. But when both parents are on the older side the task falls to the kid / kids. And when both parents are older the child / children are more likely to hear 'I'm too old' and 'we're not as young as we used to be' ... and the generation gap is big on both parts. I still think there is a bit too much romanticism in this story and many of the comments.

Commenter 11

My parents were 38 when I was born and mum has always extolled the virtues, "we have large age gaps in our family" has been said repeatedly throughout my life. My maternal granny and grandad were born in 1899 & 1896 and my grandfather's father was in his 50s when he had him. I can get back to the 1840s in 3 generation.

Now 40, I still feel I have time to have a child, especially after reading this article. My parents are both still going strong and have no major health difficulties. They had great patience but were perhaps more protective than other parents - my dad would pick us up from clubs at 3 in the morning as teenagers and claimed to love doing it! I think we've kept them young too.

How young does Ben's mum look by the way?

Commenter 12

My father was middle aged when my younger sister and myself were born. I didn't really have a sense of him being old and the only reference to it was a girl saying that she'd seen me with my grandfather at the weekend but I didn't really know what a grandfather was never having had one. My father was very fit and active and died aged 94. He and my mother were very

abusive and that is what was the issue for us not their ages. It's the type of father that you are and the quality of parenting that matters far more than being older.

Commenter 13

A really great piece. I've never really talked in depth to anyone about the complexities and regular worries I've felt having an older father. I'm 25 now and when I was born in 1990, my mum was 33 and my dad, 52. (Making them respectively 58 and 77 - soon to be 78, now.) They're both alive and, thankfully, well and as we live in the same town I try to see them at least every fortnight. I panic every time I receive an unusual call from either of them or when I can't get through to my dad when I occasionally call. (The last time that happened he'd simply dropped it down a drain... This was last month.) My dad is still a really active guy. He doesn't do as much as once did, even compared with a few years ago, but I don't think he looks nearly 80. Every now and then he looks paler than his 'olivey' skin naturally should and he seems to have a lot on his mind. I ask about everything and check all is well and he always informs me everything is fine and that he just needs to take it a little easier. He doesn't dance around the fact he's getting old and sometimes very frankly mentions that he reckons he's "got a couple of years left in him". I hate this. I laugh it off just as in the article above, but want to tell him not to talk like that. I appreciate his honesty, but I want to be blissfully ignorant. Another strange component of this is that I've often felt a feeling of guilt for putting more focus on my dad, over my mum. My mum is a fantastic woman, but as I've always felt time is more precious, more finite with my dad I've often made more of an effort. Anyway, it's really comforting and enlightening to hear of other's experiences and it has in fact made me count my blessings; as they say. Which I probably don't do often enough.

Commenter 14

Commenter 13

You're not alone! My dad was 47 when I was born and is now approaching 76. I've been afraid for years of 'that' phone call, but have gradually realised that you can't live your in fear. It's much better to enjoy the people you have around you and the moment you're in. Your dad sounds like a great guy :-)

Commenter 15

My dad was 38 when he had his first, never really thought about it until I was older as it never prevented us doing anything. It's a touch sad simply because it would be nice to have as much time with him as possible, same for everybody though I guess.

Commenter 16

My Dad was 45 when I was born, my Mum 25. The first time I was aware of Dad's age was when he fell over during the parent's race at sports day when I was 5. In my mind the fall was because he was older than the other kids dads. From that time on I knew my time with him would be precious. He was obviously aware of my concerns, death was not a taboo subject. By discussing the loss of his own parents I think that he was preparing me for the time when he would no longer be around.

He was a fantastic father and my best friend, something I am sure age has nothing to do with. He played as an active part in my childhood as any other father. He was my Dad, which ultimately is the main thing.

Commenter 9

Mmmmm all very emotional and inspirational ... but a bit more attention to the practical elements of taking care of elderly parents in young / middle age would have been appreciated. It isn't all sunshine and roses being the child of older parents.

Commenter 17
A lot of posts have extolled the virtues of having a fit and active father. I'll never know and try not to think about what might have been different in my life if my father had been, but he wasn't. He was 47 when I was born. I love him even after his death but he was tired and inactive while I was growing up, unlike the fathers eulogised in other posts. The point is that if you're becoming a father now (forgetting all the side issues) then you are more likely to be able to be fit and active, but that is still only a probability.

Commenter 18
I am an adopted child, now 48, of older parents - my adoptive family were 44 when they adopted me. My (adoptive) Mother was unable to conceive but it took many years to realise this in the 1960s, hence the late adoption. For much of my adult life I lived over 250 miles away from my parents, and was an only child, which made caring for them extremely difficult. Something that is rarely broached is the emotional and physical strain placed on children of older parents. After working 12 hour days all week being faced with a long drive to provide weekend respite care for elderly, confused and generally infirm parents really takes its toll. Don't get me wrong, I loved my Mum and Dad dearly and still miss them now they are gone. However, the loving memories are undoubtedly coloured by the stresses, strains and (yes) arguments that dominated their final 5 or so years. My main point here is consider the implications for the children when deciding to procreate after nature suggests it is not wise. No child asks to be born and parenting should be an altruistic undertaking. <awaits the troll hatred.

Commenter 19
Commenter 18
I think as others have said, there can be challenges in any scenario. I'm adopted too - my parents were around 40 when they adopted me, their fourth and only adopted child. My father died when I was six and mum was left to bring up four young children on her own, which she did through shear hard work and resilience. She died at 83 after developing pancreatic cancer and I returned from the other side of the world to help take care of her. Recently, my 93 year old uncle died after living in his own home all his life and essentially managing on his own, with regular visits from relatives/friends but he cooked his own meals and was independent. My friend has MS and is now in a wheelchair, her 10 year old daughter helps her as much as she can, and will no doubt do more as her mum's condition deteriorates. My point is that none of can know how life will pan out, who will become incapacitated and when, as well as to what extent.

Commenter 20
My dad was 20 when he had his first child (my eldest brother, 45 this year), and had two others in the following years, before divorcing and marrying my mother. They were together for several years before having me when mum was 31, and dad was 44. I am now 22 and my younger sister is 19.

Several of my friends had older dads as well, so it never really perturbed me. I knew the older siblings thing was odd - especially when I became an Aunty at 8. In recent years it has kind of struck me though, as he has recently retired at 64 and has a spate of health issues. I feel some fear that he will never really get to be an involved grandfather to my future children, and it is enormously strange to be confronted with your parents' mortality when you're only 22. Who will fix my leaky toilet or set up my computer when he is gone?!

All the same, I had a wonderful childhood, and my dad was, and is, plenty active (I suspect this was due to growing up in the country, however). He is extremely clever and thoughtful, and

people often comment on our similar looks and way of thinking. I feel extremely lucky to have a dad with such a great outlook on life and wonderful morals

... It is funny that my eldest brother is the same age as my boyfriend's mother though (he is 26 - she had him when she was 19), I do laugh at that.

Commenter 21

My father was forty-four when I was born, my mother ten years younger. I have siblings 10 and 12 years older than me, and I think my mother found it a little hard to start again with a new baby just when my siblings were starting to become a little independent. My father was always very physically energetic, however, and he loved small children -- some of my earliest memories involve trotting after him on hikes and rambles and learning from him about nature (he was a botanist). He never seemed old to me till he fell ill in the last year of his life -- a much-too-young 69. I regret that he never lived to see me established in the career he encouraged me to follow, and I wish that I had been able to have as long with him as my siblings did. But otherwise his age was irrelevant: he was every bit as much a good and loving father to me as he was to my older siblings, who were born when he was in the first half of his 30s.

Commenter 22

I have an older dad - 56 when I was born and now 82. He is 22 years older than my mum.

Growing up, my brother and I didn't really know any different. Dad has always been a very healthy and physically strong person, and still thinks nothing of putting in a day's hard manual work, although as he's got older he needs to rest more. We used to tease him when we were younger at each birthday that we'd get him a walking stick, which is funny considering he still walks unaided now. Perhaps it has helped that he has never looked his age, with people still surprised now that he isn't in his 60s, and still has excellent health.

The person I worry most about is my mum, who now 60, spends all her time looking after her own 90 year old father and my father. Seeing her experience would make me think long and hard before settling down with and/or having children with a much older man. However, they do make each other very happy and remain very much in love.

Commenter 23

My dad was 48 when I was born and my mum 37. I never really thought about their ages too much when I was growing up but have recently since celebrating my dad's 70th birthday. They live abroad so there's the odd panic if I've missed calls/voicemails from them (my dad's health isn't in the best shape) but I think that the two of them think about their own mortality, such as 'will they be able to see me graduate etc.' Anyhow, I don't think (although maybe because I don't have any other experience) that being an older parent is a detriment, if anything I feel that it's a good thing. Both of my parents retired whilst I was at school so we spent more time together which is something that a lot of my friends didn't experience due to their parents working.

Commenter 24

Really relate to this. My father was 59 when I was born, my mother was 36. By the time I was 10 in 1964 my father was 70. In 1969 I was 15 and he was 75. He never really came to grips with the 1960s and certainly never got close to understanding what a teenage boy's life was like then. We got on well without being close. Not sure that with such an age gap that was ever likely. I recall him picking up a school friend of mine for the first time who later said he thought my Grandfather had come to get him. I think that was the first time I realised how different he was to my friends' fathers.

He died in 1980 aged 84 and I miss him still and my mother in 1989. I still get caught out when I realise that people who are older than me still have parents alive today. It's an odd feeling and I wonder what I missed as well as what I experienced.

Commenter 25
My father was 45 when I was born and as a young child he could remember the end of the first world war. He used to describe what pre war Sydney was like including watching out the window of his school class room the two sections of the Sydney Harbour Bridge's arch creep slowly together day by day till they joined. As a child I was aware that he was diferent than other fathers because of his age but I wasn't able to really understand it.

Commenter 26
My father was 49 when I was born, my mother was 32. I was the youngest child of his second marriage. His daughter from his first marriage, who was 22 years older than me, had her first child when I was just two and her second when I was four. It never seemed odd to me to have such an old father, though I was aware he was the age of my friends' grandfathers. In those days, all fathers seemed to be emotionally remote from their children, plus my father travelled a lot on business and was, by nature, a quiet man who kept his counsel. As kids, my niece and nephew and I had great fun making people guess our relationship. Today, I just find it too complicated to describe and say they're my sister's kids and my cousins. The big transition for me was from regarding my half sister as an aunt to appreciating her as a sister, a move she initiated and kept at till I came around as a young adult.

Commenter 27
Thank you for writing this article. I've really enjoyed reading it and the comments. It’s so nice to hear from other people in similar positions. My Dad was 52 when I was born, and my Mum 39. As a young child I was never really aware of it. He was always so active, doing endless DIY projects and gardening after work and during the school holidays. I remember once when I was at primary school and he came to pick me up from ballet lessons, my teacher mistook him for my grandfather. That moment has always stuck in my memory, as up to that point I had never noticed any difference. There are many things I am grateful for in my Dad; his strong belief in education and his pride in us, his children. He loved to tell his friends and neighbours about all of our achievements, which embarrassed us to no end! He was such a sociable person and loved meeting new people. He also loved talking about his local area and was passionate about history and poetry. When I was in my early 20s he was diagnosed with vascular dementia. We had noticed it coming along gradually, but it was still a shock when we knew for sure. My mum became his full-time carer, to the point that she couldn't leave the house for more than an hour or two without him. I felt guilty for not being home more often and helping out. In some ways I resented having to deal with issues like this, when I was still so young. Most of my friends' parents were still working and living full lives.

My Dad passed away in 2013 at the age of 81. He never forgot who we were, but he had begun to live in the past, where his memories were clearer. In some ways it was a blessing that he passed away when he did. It has got to the point where my Mum could not take care of him on her own and we were having to consider putting him in a nursing home. None of us wanted to do this, but my mum had no real life to speak of, other than looking after him. Thankfully this never had to happen, and he spent every day, up to the last week, in his own home.
I know there are many benefits to having an older parent, and that illness can happen to anyone at any age, but nonetheless, I don't think I would even want to have children at that age. I read an article recently that said research has shown that children born to older parents are more aware of their mortality and tend to worry more about it. In my experience that is very true. I

loved my Dad, but part of me feels like I missed out on a lot. I see the relationships that some of my friends have with their Dads and it makes me sad that I will never have this, and that his grandchildren will never know him.

Commenter 28

Really interesting piece my own father was 48 when I was born and was a brilliant and loving dad. I became father myself at 44 after not thinking I would have kids myself, I just hope I can be half the dad I got. He passed away 12 years ago today.

Commenter 29

External commenter

Yes there is a generation gap but somehow there are many other qualities that make up for that. My father was so grateful to have children later in life- I think he believed it would never happen- that he made sure he gave his all to his family. I loved him very much, despite adolescent arguments.

Commenter 30

My dad was 69 when I was born. He died eighteen years ago, when I was 17, and he was aged 86. I miss him and I'm sometimes sad that he isn't around to see what I've achieved, but I'm incredibly grateful that he (and my wonderful mum) had me. I'm very glad to be here and I like to think he'd be very proud.

Commenter 31

I had older parents, as did my two youngest kids.It's not always about choice, either. I was born after WW2, which prevented my mother & father from starting their family sooner. My mother was 36 when I was born, 38 when she had my sister, and when we were kids there were a lot of other families in similar circumstances. The sad part was my fathers death in his early 50s, when I was 30 and my sister only 18, but he obviously died rather young.
I had my first child at 20 and another 2 years later, then a great big gap before my third and fourth were born when I was 36 and 38, respectively. They were both splendidly healthy babies, have grown up as impressive women, and I don't think my age affected them badly. When my son had his first child, my youngest was 6 which caused a few giggles when she insisted upon her proper title of "auntie". I suppose really the concept of "generations" gets a bit blurred by so much overlapping, but then it probably always was. Anyhow, youth is certainly no sure protection against children being orphaned while still young.
As a young mother, I had more energy but less security and money. As an older mother, I had more patience and confidence, a better washing machine and my own home.

Commenter 32

I had older parents. My father was 30 when WWII (US) started and he didn't meet my mother until after the war. They married at the end of 1951 and I was born in 1953. My mother died due to breast cancer in 1958 so I have no siblings. My mother being deceased was odder than my father being older. Everyone had a mother and a father as divorce was rare even in the US at that time in my area, so not having a mother and being an only child made me and object of both pity and overly concerned attention. I had cousins that could have been my grandparents. It was a little strange, but for the most part I didn't spend a lot of time thinking about it unless it was pointed out. My dad was my dad and I loved him and he loved me and that was all that mattered.

Commenter 33

My mother was 39 when she had me. Not terribly old in comparison to many of the stories, and ow she's 75 and although i'm still happy with the freedom that means I could decide to have

children at forty or so, I do remember growing up feeling isolated from others, as my mother was from a different generation to everyone elses, so couldn't relate to me on so many levels. She's only ten years younger than my grandmother (my father is ten years younger than my mum) and now the two matriarchs in my life are nearing death whilst my friends parents still have lots of life left. I love my mum more than anything, but I wish I had the chance to have her a little longer.

Commenter 29
My Dad was58 when I was born. He was kind, loving and made sure he had time with me everyday. My sister was born when he was 64. Sometimes I didn't appreciated his old fashioned attitudes but now I know that he instilled great values in me. I only had him for a short time but I was so lucky to have such a great Dad

Commenter 34
My Great Grandfather was born in 1825, my Grandfather in 1864, my Dad in 1901 and me in 1947, and my daughter has just had her first and only child at age 43. It's not a lot of generations in 190 years. Probably one of the pluses was that I was only 40 and able to look after my ailing father when he was 86. I always say to people, leave having the last one till you're older so they can look after you. It works.

Commenter 35
My dad was 54 and my mum 48 when I was born. My brothers are 20 and 14 years older than me. My dad died aged 95 and my Mum is now 94 and still alive. I had loving parents but chose to have my own kids in my 20s. Now in my 40s I'm glad I did as my kids are independent whilst I'm having to be a carer for my Mum. Very young children would make that impossible. My parents were fabulous, hands on grandparents and if I'd had my kids in my 40s that wouldn't have been possible, I guess there is no right way but I think younger is certainly easier

Commenter 36
Brilliant, beautiful story.

There is the issue of 'generation' squeeze which happens to children born to older parents, who then have children late themselves. Like me with an elderly Dad 82, and a little son, just started school. I deal with both ends of the life span - which keeps me busy - but is also rather wonderful. One thing is sure, older grandparents are not going to be so involved with grandchild care - I have never relied on my parents for babysitting in regards to my work due to their age.

Commenter 37
My parents had me when they were 42, in 1981. My mum loves telling me that she was the same age as many grandmothers visiting their new baby grand children on the maternity ward. She met my dad at 37 and I don't think she ever thought about the age differences between her and my friends mums and dads - when I was growing up, and even now, I was the only child with 'old' parents. Mum and dad always seemed young for their age, and only since my father's recent death have I felt deeply sad he didn't have me at a 'normal age', the vast majority of friends my age haven't experienced an ill or dying parent, so it feels quite isolating. However, I wouldn't have changed him for the world!

Commenter 38
I have to say that my father had me when he was 43 , my younger sister when he was 45 and my older brother two years before myself and I see that many of you were embarrassed my older dads or mothers but I've always felt incredibly proud of him , he raised a family that wasn't his biologically 20 years before he had us, he is a nurturing and loving man , he's 64 and I'm 17 but he still always finds ways to have fun with me and my siblings even my half brother from

my mums first marrige (she's 48) he was diagnosed with a disability when I was around seven but it's never stopped him from taking us to parks or being a good father , I think the fact that most older parents have life experience before they have children is a benefit and we should never lie or be ashamed of the people who have made us

Commenter 39
I was the youngest and born when my parents were older. They were always healthy and energetic and still pretty much are. No problems. Not everyone has health problems.

Commenter 39
One thing I have to say, there was a generation gap in culture. They liked old style classical stuff, completely skipped over the whole modern culture. No rock music, hate modern art, hate modern movies. But I think that has as much to do with their personality and upbringing than their age, too. But it didn't bother me too much, because I like the old stuff too. It was good to have a taste of both worlds. Plus they're are on the liberal side too, so they like what they like, and they let me like what I like.

Commenter 40
I must be an exception, I did not enjoy having a father who was in his 60's when I was born! My mother was 35 years younger. My mother died when I was five. The other kids at school always though he was my grandfather. He was born in the reign of Queen Victoria. Always an outgoing child, I suffered from his elderly intolerance!

Commenter 41
Thanks for sharing this. My dad was 17 years older than mum; I was born when he was 54. He died at 65, after suffering for 3 years, largely at home, and terribly at the end, of prostate cancer. He refused hospital after initial treatment, and would not discuss his illness; mum didn't actually know what he has until after his death. Which says something about a generation, attitudes, and her role and approach. I never got to know him properly and those years were overshadowed by illness and fear; that affected me hugely, I was on a different planet to my contemporaries and to an extent still am at pushing 50. My brother, 8 years older, bore some of the brunt of physical care and I know was deeply shocked. I have some good memories but not enough and miss what could have been. I appreciate these situations are no-one's fault or ultimate responsibility but older parenthood does increase the risk of losing parents early and can increase the difficulties and stress inherent in parenting and family life.

Commenter 42
My father was 50 when I was born and died 2 days before my 21st birthday- that was 30 yrs ago. My eldest daughter is 21 in 2 months and it brings back all the feelings of despair that my father never saw me graduate, marry or see his grandchildren. I'm still quite envious of friends and colleagues who still have their parents (and even grandparents!) still alive

Commenter 43
well great, but what if he'd suffered from dementia or arthritis while they were small? my own father was 60 years older than me and that wasn't much fun at all, as he simply didn't have the energy

Commenter 43
External commenter
as someone whose father was 60 a few days after i was born, i think i have a bit more experience to rely on than yourself, mate. i suppose it's a bit too late for you to try growing up with a father who, because of age, lacks the energy to keep up with small children... it's no fun. he suffered

from prostate problems, arthritic hands, and his memory wasn't all it should have been, so i reckon the 'say nowt' bit still stands

Commenter 44

External commenter

You can get ill at any age. Older age does lead to some illnesses more but is not a definate. From my own experience of having a older father I've seen how being surrounded by the young tends to keep you young and active.
I've also seen plenty of 20 year old who do nothing but dump their child in front of a TV instead of engaging and informing as my father did with me.

Commenter 43

my own father was 60 four days after i was born (as the youngest of seven) believe me, whichever way you turn it, it's not much fun having a dad that old. i'mm 55 now and just ended a relationship with a wonderful woman of 40 who wanted a child more than anything - but to be, say, 65 when your kid's ten? parenthood is demanding enough without adding the problems of ageing... and i remember the strange atmosphere and funny looks from my own childhood whenever i went anywhere with my dad, which didn't happen often as he was just too old and decrepit

i wouldn't wish that on anyone

Commenter 45

Commenter 43

My mother was old when she had me and, as you say, I wouldn't wish that on anyone.

Commenter 43

Commenter 45

i hear you. my own mother was past 40, and the stress of another child with a husband of that age was enough to break her already fragile sanity. it affects everyone when someone doesn't have the energy to run a family - and as a result, many of my older siblings had to take on a carer's role, which they completely resented. i can imagine other families being more caring but, as the Danes say, when the manger's empty, the horses bite

Commenter 46

Commenter 43

Yea it sucks huh. I felt it made me grow up too soon. My early teens were filled with hospital visits and late night vigils.... Am glad I had my daughter when in my mid 30s so she had the full benefit of me and her mum in our prime...

Commenter 47

External commenter

My father was 50 when I was born. He is now 85 and living actively and independently. My mother died in 2007 at the age of 60 from cancer in the same year she was due to retire. You can never tell how things are going to turn out so just live for you child and don't waste time worrying and questioning it. It is what it it is, just make sure you are as healthy as you can be... and enjoy it!

Commenter 48

External commenter

My father was 50 when I was born (my mother 21) . . . I had similar annoyances growing up, such as my dad getting tired kicking a ball around after a short while or getting embarrassed

when friends from school asked about grandpa, however I wouldn't change it. He's a great father to myself and my younger brother (he didn't stop with me!) and my two, much older half brothers. Plus, I get to hear stories about growing up in WW2, about seeing Puskas and Di Stefano play live and other invaluable life experiences. . . so its not all bad!

Commenter 49
External commenter
My dad had me at 55 and he has been and still is an absolutely wonderful parent. He was lucky that he has had very good health, but to be fair to him, he has worked to keep it that way. He used to run marathons and still now goes running several times a week, usually accompanied with a 'Oh, I really don't feel like it tonight'! I understand and can identify with this article, but I also could not have asked for a more wonderful father. He had so much life experience and wisdom to pass on to me. I wish you the very best in your new life chapter of fatherhood!

Commenter 50
External commenter
Agree totally. Now he's an adult with some perspective on it I'm amazed he's not more explicitly resentful towards her, I would be. My Mum was 41 when she had me, and brought me up on her own. I clearly remember one time we were skint and she was having a good moan about money, and she finished off saying something like 'It's so much better now you're older and I can talk to you about things like money problems.' I think I was about 8. I think sometimes single parents/one of a couple who has to care for the other must feel lonely and frustrated at not having an adult to consult and confide with, and their child becomes a substitute. And my example is obviously nothing compared to what the author's gone through, his mother's lucky he didn't bolt out the door aged 18 and never come back.

Commenter 51
My mother was 44 when she had me, not too old by today's standards but I do wish people would very carefully consider having their children earlier, when they are more likely to be healthy until their children are much older.

I have slid into caring, gradually and without really noticing. Since my mother was in her early sixties, her health has been poor. By my early twenties, I was assuming responsibility for her medication, and helping her deal with diabetes, and arthritis, and a host of other problems. Now, fifteen years later, she also has dementia (although thankfully a very mild form) and I am still caring for her. She needs constant reassurance, and i am struggling, imperfectly, to provide it.

I wouldn't change my parents, but i dearly, dearly wish they had had me earlier. My siblings were born almost two decades before me, and had twenty extra years of healthy parents. They knew my father when he was healthy and had the chance to grow into adulthood before he died. They had the time to forge careers and form their own families without having to compromise to care for our parents. In contrast, I feel that I made an almost unconscious transition from child to carer, and my own life has been on hold. I have managed to get a degree, and i have held down a job, but I am reaching a stage where I may have to make a choice about whether to care for mum, or continue to work. That won't really be a choice for me. Work is work, but family is what matters. But i fear for the future.

I identify so much with this article, particularly it's mention of stigma and the indignity of having to share personal information with others. Mum leaves messages with the receptionist at work, and i have to make calls to her and about her from my phone on an open-plan office! And there is a stigma: to many people I am simply a man in his late thirties who still lives at

home. Few people resist the chance to judge me harshly on that, and if i tell them that i am a carer, contempt frequently turns to pity - I am not sure which is worse!

Commenter 52
Definite downsides to having an older dad. Maybe I should be grateful that my dad (69 when I was born) ran off with another woman while Mum was pregnant with me and never visited. I was spared embarrassment and heartache and have a potentially randy old age to look forward to!

Commenter 52
With reflection feel bad for posting that jokey response. My dad was an absolute shit and died suddenly while in good health at the age of 99 - so we could have had a relationship well into my adulthood if he'd wanted it. sucks that you had to go through what you did - life really isn't fair.

Commenter 53
Feel very sympathetic. But there are lesser stories to make the point. When I was in my teens in the 60s (yes the major change of the 20th century) my parents were pensioners and totally out of step with the times. When I was 21 my mother tried to sabotage my holiday with my girlfriend (now my wife). She rots in hell I hope.

Commenter 54
My dad was 49 when I was born and died suddenly after a short illness at the age of 66. I wish I could have known him as an adult.

Commenter 55
I had older Parents. Not super old but people still had kids in their early-mid twenties at that time so they were older than most. I soon noticed they were more in step with the previous generation than some friend's parents.

Obviously a different league to this man's tale but I really think this grand social experiment to 'hitting 35' (if a woman) or even older if a guy before having a kid or two has more downsides than upsides.

Commenter 56
My dad was 62 when I was born, but contrary to the one described above, he was very healthy. It took time for me to realize the downside; the lack of synch between what went on at home and what went on in my friends homes, etc... Also being the son of British expats in Brazil there were further reasons to feel alienated. Not too sure if I ever digested the issue properly, all I can say is that we lived in worlds apart, yet under the same roof.

Commenter 57
External commenter
But what if you haven't met the person you want to have children with until later in life?

It is only by confronting and challenging such close-minded attitudes that they will change. Bowing to them by not having children will only perpetuate them (and I speak as the daughter of an older mother). Many of my friends have had children later in life and their children are having a wonderful time.

Commenter 57
External commenter
I agree. There's no imperative to have children. But no woman is when they're 52. Mother Nature works in a way that's best for nature. So if a woman doesn't meet the man of her

dreams, is healthy and able to bear children, why should the opinions of young children stop her? Des O'Connor once asked why he should deprive his wife of the joy of having a child because he was in his 70s and I tend to agree with him.

Commenter 58
I was 23 when my dad died at 66 years old. I still miss him, but he was a great father. My only sadness is I didn't know him long enough and my son didn't hv a wonderful grandfather, just a useless father as a role model.

Commenter 59
I married at 21 and 6 months later my husband was stricken with MS. He wanted to have a child before he became too disabled. I refused. I cared for him for 11 years, and when he died I was 34. So many people approached me afterwards and said, "Well, you'll want to find someone else and have children right away..." I would just look at them. Oh, yeah, right; like I need to be taking care of someone else. My parents were 41 and 45 when I was born, and I always felt out of sync with my peers as well. I will died childless and content to be so.

Commenter 60
Really interesting . Must have been so hard. My dad was mid 40s when I was born. Not quite the same but I relate. When I was 16 dad became terribly ill with cancer, so me and mum assumed a caring role

Commenter 44
I'm fizzing mad at this but also sympathetic to the writer for their plight.
My own father was also 64 when having me. Mum 28 years younger. I have a brother and sister the same age as my mum and one sister only 6 years older than me.
My dad was a wonderful parent. His attention was garunteed and I thrived under it. Yes I also had people ask about why I was going out with 'grandad' but it taught me to not care about others opinions of my life. Sadly my dad passed away 4 years ago at the grand age of 90. He had cancer the last 3+ years but maintained dignity even with failing hearing and need for help. I love him and miss him daily. But I would never suggest anyone older would be best not having children.

Commenter 61
My mother was 45 and my father was 54 when I was born. I wouldn't change it for the world. They were the most competent patents ever.

Commenter 62
I have really mixed emotions about this article. I really identify with the writer,although my mum was 41 when she had me and my dad was early fifties. I had a great childhood but my dad started to show signs of Parkinsons when I attended secondary school.I remember other children laughing at him in the car when he had to wear a neck support because his head was shaking so much.He used to pick me up from school.I remember my mother's clothes always being more ahem mature than other mothers,small things you would notice that differentiated you from the rest.

My dad died when I was 24 from complications arising from that horrible disease. I'm now 39 and have been looking after my mum who has dementia for the past three years full time.I am the only child so the onus is on me,she was in a home but didn't want to stay there,so I moved back to my hometown to help her.The guilt was heavy in my heart.I know I made the right decision but the future is scarey to think about. I understand people having later births but it is not for me I think. It is too much of a gamble and even though I am a man,the heaviness that

lies within as you make choices about your own life, while knowing your parents are sick or getting more fragile is for me,too much to ask of another.

Commenter 63
My mum and dad were both older than usual when they had me - I'm 27 and my parents are now 74 and 62. I do remember being a bit embarrassed about this when I was about ten but I don't think it was any more of a problem than all the embarrassing things my friends thought their parents did. I was never more bothered by their age than my friends were about things like their parents dancing or awful jokes.

They aren't in the best of shape but people with younger parents have had to watch their mums or dads going through cancer or serious disability whilst mine are just a bit tired - age isn't the only indicator of how likely you are to get ill. And their health, when it has been bad, has never affected me or my brother.

They are two of my best friends and I wouldn't change them for the world. If anyone reading this is an older parent themselves or is considering being one and feels scared by some of the negative stuff above, please don't be. Older parents are no better or worse than young parents. The most important thing is the love.

Commenter 64
My dad was 50'when I was born. By the time Inwas 30 we had next to nothing in common, but that could have been because he was an abusinve, cantankerous drunk. But o suffered miserably in childhood with a Dad who behaved as if it was still the 1920s.

Commenter 65
Like Kate, my dad was 57 when I was born, and my mum was 28. He never really looked his age, was very handsome, and for years we always thought he was much younger than he was. I never consciously feared his death, it didn't occur to much during childhood - and in fact he lived to be just short of 90, dying when I was 32. I had friends whose younger fathers had died before him. I don't think it's age that is the problem, but fitness, and attitude. My dad was very Victorian in his attitudes - because he was a Victorian. But I loved talking to him about his youth, because it was living history! He was lucky to make a good marriage with my mum, but having babies later and later will no doubt throw up some interesting challenges. If this interests you, you might like to read my novel, just out, which is about this topic. It's called Larry's Womb, and here's link where you can read about it - and read the first 40 pages. https://completelynovel.com/books/larrys-womb

Commenter 66
My father was 48 when I was born. He died in 2009 at the age of 86. I thought about it once and a while, but, it never really worried me. Far more importantly, he was simply my dad: a brilliant, loving, kind, and generous man.

If you give love, your family will feel love. All this future casting is nonsense. Any of us could go at any time. Give love now, and age or how long you have together doesn't matter, it could be over tomorrow anyway, no matter how old you are.

Commenter 67
When I was born, my father was in his early 50's, and my mum was 42. They were both younger in outlook (mum) and appearance (both) than many of my friends' much younger parents. I never felt embarrassed about them. I adored my mum. The sad thing was my mum died when I was 22, and finishing my degree, and my father died a year later. Mum died of cancer, which could have happened to anyone, and my father was a

heavy smoker. I did feel a bit alone in the world as I have no siblings, but I have good long-term friends who are like family to me.And now it is them who envy me my lack of parents, as theirs are ailing and getting dementia. Swings and roundabouts. I don't think we can generalise about the ages of parents - some parents are good and some bad, it's not to do with age, in my opinion.

Commenter 67
External commenter
That's true. I had just one grandparent living when I was born, and she died when I was 8. I was a bit envious of kids with grandparents.

Commenter 68
My Dad was in his 50's too. The only thing I'd add is how much more prepared for his death at 83 I was. I'd been preparing since I was a teenager and he had a stroke. It's odd to go through your teenage years expecting you father to die.

Commenter 69
My dad had retired well before I finished school. All around me fellow pupils seemed, through their parents, connected to a welcoming adult world (I admit this was, on my part, a bit delusional).

I ended up basically forcing myself on younger blokes - substitutes - because my family seemed so isolated from what was in store for me.

Later I ended up spending 10 years caring for my parents as they succumbed to dementia, heart failure etc... I don't wish my experience on anyone, though of course I loved my parents and miss them.

Commenter 70
I have experience of both being a child of an older parent and an older parent of a child.

My father was 50 when I was born. It was a disadvantage as he didn't have much energy or enthusiasm and there was a big generationally gap (his mother was a Victorian and that showed through in a lot of his behaviour and beliefs). We didn't have a close relationship and at least part of that was due to his age.

I wasn't as old when we had my daughter but still much older than first time parents in general. When she went to her first school there were parents a lot younger than us but in her present school (private) most of the parents are relatively old so we don't feel any different.

I do worry about not being in my daughter's life for long enough but on the other hand we have financial security and I have more time to be with her. We have a very close relationship and I just hope that continues for all the years I have left.

Commenter 71

My mum was 45 when she had me and my father was 50,this was in the early 70s,and the kids at school always used to ask why my Gran picked me up from school,a little embarrassing for a 10 year old.My dad died when I was 10 which is a definite negative about having older parents, my mother is nearly 90 now and housebound and I love helping her out, she is a very cute older person.My parents were settled and stable when they had me,but I've got 4 older siblings(my youngest sister is 13 years older then me),and I did feel like I missed out on family occasions when they were all young.

Commenter 72
External commenter
I enjoyed standing on my own two feet for the brief time I lived overseas! It was incredibly freeing! Part of me would absolutely love to be responsible for no-one but myself.

The point about elderly parents is that they *don't* necessarily die young--and you as the child spend your teens, 20s, 30s, looking after an increasingly frail, dependent, person who is by the expectations of society (and the NHS) entitled to your care and devotion.

Who's selfish?!

Commenter 73
As someone with older parents I do sympathise with some of these comments. However, my mother had me at 40, so she was a fair bit older than my friends mums, and NO ONE, not even once whilst I was in school, made a negative comment about her age. In fact, most of them were surprised and impressed when they learned her age.

I'm now 26 and she is 65 and still working. Her age has never once had a negative impact on my life, quite the contrary as she has so much wisdom and life experience that she shares with me everyday. She is the most vibrant, energetic and life affirming woman I have ever known. Some people commenting have said it's sad that people with older parents will only know one generation, but my sister (who is 9 years older) has 2 children and my grandmother is still alive and about to turn 90. So even with an older parent, there are regular occasions when we are 4 generations in the same room. At no point in my life have I felt it was selfish for her to have me at 40, or has it negatively impacted my life.

Commenter 74
My dad was 42 when I was born, which is not particularly old in the context of this article. I remember when he was about to turn 50, it dawned on my sister and I that he was getting "old." As children it was at about the limit of our imaginations in terms of decrepitude.

I'm 26 and my wife and I are expecting our first child. My dad has assured me that bringing up young children was one of his favourite experiences. But I wonder whether he was any better prepared for the experience, having had an extra decade and a half.

Commenter 72
External commenter
Actually, while most people start their 60s healthy, most people (even the vegan runners) enter their 70s with a major diagnosis of some sort, which has an impact on their family.

And while the rates of dementia appear to have stabilised in western populations, I think that the point at which people are diagnosed in their 70s-80s masks the fact that they've had cognitive impairments for *years* before that; that's not just my experience with my father, but with all of his contemporaries. I suspect it's hugely underdiagnosed, especially when the impairment isn't with memory, but with subtleties of judgement and mood.

Commenter 75
My father had me at 55; I am probably not the best example for this article as I have found the age gap between us has made us more distance rather than closer. We do not have an amicable relationship and I find myself only staying in contact with him due to his age. He is about to turn 80 this month, I find myself thinking about his mortality more as the years go by. I feel that due to our mutual dislike having the responsibility of him in old age will make this worse.

I think maybe if he was different it might be another experience. But I find his age related racism, mistrust of technology and bigoted behaviour hard to tolerate.

Commenter 76
My father was 45 when he had me - which is a bit younger than these examples, but a positive to this is that he had a wealth of life experience and as was able to retire around fifty I had a full time Dad which was excellent. Sure there are drawbacks but also a lot of benefits.

Commenter 77
I found it tough. My dad was 55 when I was born. I hated the other kids asking if he was my grand dad. We never really went to the park for a kick about or engaged in physical activities together, there was always a terrible obvious generation gap, he was very critical of my clothes, friends and other lifestyles choices through my teens. He grew up in a time when teenagers didnt exist. He died when I was in my early 20's. I wish I could have gone for a pint with him and get to know him as an adult.

Commenter 72
External commenter
Me too! That's what I miss--being able to just know him, as an adult; have a coherent conversation that isn't part guess work from the aphasia and dementia, relieved from the need to keep an eye on his meds.

Commenter 72
I'm the anonymous poster above: Guardian editors, you really twisted what I said.

Among other things, the edited-out-of-existence point of the second paragraph is that I am *not* a member of the so-called sandwich generation--they are people who, from my perspective, have it all. I have a good job in administration, but it's not a career, it's a job that pays the bills and allows the flexibility I need to care for my parents. I have been a carer since I was about 12; so, no career like my peers, and I most certainly do not have a partner or children of my own.

Secondly, while my parents are an unusual case of older parents who lack financial security (as in, debts and assets equal zero and their annual income is well below the median), money cannot fix everything and it's rather sad that several of the older parents here are citing financial security as a good thing, a benefit to, or reason for, being an older parent.

Certainly, life would be much easier if I could pay for nursing care for my dad, but wouldn't change the fact that I've never known my dad when we were both functioning as adults. He began exhibiting the paranoid behaviour, impulsivity, forgetfulness, and mood swings which signal the beginnings of dementia when I was 11 or 12. I have been managing those symptoms and their impact on our family since that time. He was officially diagnosed when I was 27, after fighting with my mother for over a decade to have him assessed.

Grieving for a living person with dementia is hard; it's harder when that person still feels that they need to be parenting you, and when you yourself are 12, or 18, or 20-something, and really do need parenting. It's hard when you are both fiercely protective and proud of your dad, and fiercely jealous of everyone else who has parents to talk to and rely on for emotional and social support. That thing I "feel horrible about thinking", which the Guardian nonsensically moved to the first paragraph? I've been envious of people who have lost a parent when they were a child, teen, or 20-something, because you see other people in the community step up to help. My parents are still "there", so no one helped--I've felt utterly alone at times, and have been

almost solely responsible for the well-being of my younger sister since I was 13 and she was 9. She and I now have an adult relationship that I do not have with our dependent parents.

I love my family. Fiercely. But I think having a child in your 50s is as irresponsible as having one in your teens--and less forgivable, as an 50-something really should be able to think through the consequences. So, if you already have a child more than 45 years younger than you are, talk to your child openly about the issues that they'll face, and ensure that they have a half-sibling, a family friend, someone 20 or 30 years their senior whom you both trust to help out (spouses are bastions of denial; it's not right to make a child challenge both of their parents' mental fitness). And if you are considering becoming a first-time parent past age 40 or 45: Don't. Though, since I'm human, that does not mean that I love my parents any less.

Commenter 72
External commenter
"You can't extrapolate your own situation on that of other people"--of course not, but neither can you, and we can both look at the data we have. I can look at the data from the 1946 Cohort study which shows that its fit and healthy members who grew up with modern medicine and the NHS, had, on average, two major diagnosis by the 2010 follow-up, and only 1 in 6 were in 'good health'.

Life expectencies are expanding because we can manage those major diagnoses, not because they are not happening.

So no, I cannot extrapolate, but there is data to suggest that the idea that 'sixty is the new forty' is somewhat naive.

Commenter 78
Commenter 72
Well said. I'm in a similar position, though not as extreme as you in terms of age difference. My mum had me when she was 39, which is relatively young compared with the stories above; but my dad, who was younger, died at only 52, and I'm an only child.

Mum now has end stage dementia - a condition that has been coming upon her for the best part of twenty years. Like you, relationships and family have passed me by, due to the escalating long-term demands of caring for my mum, who has never been aware of her increasing incapacity.

She is now 100% dependent in 24/7 residential care, but as we are each other's only close family, I remain responsible for her welfare, although she no longer knows me (which is emotionally very hard, given that I have no-one else who remembers my lifetime).

Of course this situation can still arise when people are not so old when they become parents, but the odds are obviously far greater if you're over 40. Older parents, when interviewed, always seem to focus on the perceived disadvantage to children when they're minors, but it's when you're a young adult and your parents are already elderly, frail, and dependent, not middle-aged and still active, that the age gap really starts to impact.

I know exactly what you mean about envying the "sandwich generation", which has those two slices to make a sandwich! I'm now 50, my career is in ruins and I have no-one, after many years of seeing mum deteriorate and fighting to keep her safe and well. I wouldn't wish that on a child.

Commenter 72
Commenter 78
We're not alone! We just have to talk more about our experiences! And, I think, not be afraid to criticize the societal expectations that mean it's seen as OK to have a child as a 50-something. That point about the odds being far greater for a child or young adult to be dealing with a parent with dementia if the parent was over forty at the child's birth is SPOT ON, and glossed over by so many of the discussions around older parenting--so too is the point that families like yours and like mine are so much smaller now, with one or two children and few if any cousins, meaning that the caring falls to one person rather than being a shared responsibility.

Wish I could make you a cup of tea!

For what it's worth--my area has volunteers who work with foster children, and accept older foster parents, too. You probably have an amazing capacity for caring and compassion, so maybe there's something similar in your area, down the road?

Commenter 72
External commenter
"Bloody hell. Too late now. I'm 52 and me and my Mrs have just had a daughter! What am I going to do now? I can't send her back."

And I hope that you'll have decades together! I did actually answer your question, even if you meant it sarcastically--it's really important that your daughter has someone (or a few someones) outside of your immediate family who can check in with her and with you, so that when she does start having to care for you, she has back-up.

You may not need help until you're 95. But it's more likely that you'll need a bit of a hand with things starting when she's 18 or 20--kinda hard to quietly look after your dad while you're simultaneously at uni, trying to work two jobs, and maybe find someone to start your own family with (and, she's a daughter: she has far less time to start a family than you did).

Commenter 78
External commenter
As Commenter 72 says somewhere below, make sure your daughter has plenty of other people around her as she grows up, and encourage her to spend at least as much time with her peers as with you and your wife.

Don't worry about the superficial "do I look old at the school gates or unfit on sports day" issues; those don't matter. Whereas making practical plans for your old age (power of attorney, care choices, finance), building a wide support network, maintaining your own emotional independence and supporting your daughter to develop hers are positive things you can do.

I realise that's probably not the serious answer you wanted, but hey-ho: life does gets serious! And you are the adult here.

Commenter 72
External commenter
1) Ask anyone who has dealt with a parent with dementia of any age: spouses are usually deeply in denial about the situation (at times, and depending on the type of dementia, the spouse seems less aware of the situation than the dementia-affected person). So, it usually falls to a child, sibling, friend, or co-worker to broach the subject and negotiate the early stages at the very least. The second part: it *is* related to her age, and that of any couple with a significant age difference--they've already demonstrated unusual social judgement in selecting their spouse.

Both of my parents (and many unusual couples) are romantics, and idealists. Not helpful when dealing with the bald practicalities and frailties of ageing.

2) Yes, dementia is terrible for the sufferer and their family no matter the age. You ask would it have been easier, had I been 10, 20, or 30 years older at each point along his decline. Bloody hell. Yes. I was 12--an age when most kids are just realising in a coherent way that their parents are merely human, and not magic superheroes, when I realised that my dad's judgement, memory, and coherency were off in a way that made him tremendously vulnerable, and since he dealt with all of the practical financials etc. in my family and my Mum was unwilling to see it, also made our family tremendously vulnerable. By the time I was 17, his driving had deteriorated to the point that I would simply intervene and drive my family anywhere, with the excuse that is was 'practice' on my newly-minted licence. Not awesome when you're also trying to do school, uni, a job, and figure out who you are. When I tried to articulate my concerns to *any* adult, and *any* point about *any* topic, it was dismissed as the moanings of a typical teenager. Which I took to heart, and decided that it was *my* judgement, and *my* powers of observation which were in error.

Would it have been easier to cope with that, at 22 (Dad late 40s, instead of late 50s)? It would have been hard, but I would have had a dad (and mum) to rely on in my teen years, and perhaps more faith in my own observations. It would have been rotten to suddenly have to deal with that, after having a relatively normal adolescence, but I think I would have been significantly better equipped. Plus, I might have had a more normal relationship with my sister; a less intense responsibility.

Would it have been easier at 32 (Dad late 30s)? Since I've started to hear a bit about other people with parents beginning to deal with age-related illness, since many of my sensible peers have careers and partners; yes, while it would have been a little early, and maybe a little stressful if I had been hoping (like many of my peers) to have my parents do some of the childcare for their grandchildren, having those extra 20 years to develop my own life and identity would have been brilliant.

Would it have been easier at 42 (Dad late 20s)? I'm crying writing this. It would have been really, really awesome to have had a couple of decades of adult life able to trust my dad (and my mum) to look after themselves, at the very least. I know from other people who my parents were, earlier in life, and I think that they would have been the type to provide unconditional back-up while I try to figure out all of those things--work, love, home, that seem to pre-occupy people. And, I don't have kids and I doubt at this point I'll have my own, but, if I'd been 42 and not 12 when the dementia kicked in--presumably I would have had 5 to 15 years of childrearing time when I could call my own parents with questions, and trust the response.

Having an aged, frail parent is not at all like losing a parent young. If a parent dies early, of an illness, often they'll have a sibling or friends or coworkers who know what's going on and can help out the family, ideally, and once the parent has died--you're free to look after yourself. There's none of this decades-long gentle decline, where the parent is only sort of ill, and in order to maintain their dignity (and yours), you have to do the caring in an invisible sort of way. I have talked to other family carers of dementia patients. Mostly, I try to empathise with their challenges of dealing with their parent, their career, their spouse, and their children (who are usually my age or a little younger) and grandchildren, but sometimes it just makes me feel far more alone.

So, yes, dementia is a tragedy for all involved at any age. But adding 10, 20, or 30 years to my age would have helped immeasurably at each milestone.

Commenter 79
My Mum had me at 43, in 1974. I used to get teased at school for having a Mum over 50, which I found really hard at the time. People always assumed my parents would be real disciplinarians because of their age, but they couldn't have been more kind and laid back. I wouldn't change a thing, and I would hesitate to have another child myself (in my 40s) if I could afford it!

Commenter 80
My father was 40 when he had me and mom was 38. This was 55 years ago. He died when I was 26 , mom died when I was 9. I spent some time in a foster family while he was in hospital with TB. As well as living with two other uncles at different stages between being 9-13. So I never had a proper childhood in that sense as he was always chronically ill till he died aged 67. We lived on social as he was retired through ill health at about when I was 11-12. Of course I miss him but I don't miss the way we lived. I myself became a father at 43 so , sure I´m worried about health & losing my job. But at least I have both of those for the time being. I often think I´m too old to cope but there are men 10 years older than me who have babies, and you see all those celebrities marrying women 25-30 years their junior & knocking them up & you think WTF ?

Commenter 81
I deliberately had children younger than my parents - and even so I worry that my mum will not live to see my children grow up. My dad died suddenly, having been a grandparent for three years.

There are no wrongs and rights, but it is nice to be able to keep with with your kids. And to think that you will see them grow up and will know their children (if they have them).

Commenter 81
External commenter
But that's actually not necessarily true. Older parents aren't necessarily more patient - it's quite difficult to adapt your way of living a age 44 than it is at 28, for example. And if you honestly think people in their 40s aren't ambitious - take a look at the CEOs and politicians. Similarly, young parents might hate kicking a ball in the park. Or might not have time as they're working three jobs to make money.

Older parents are far more likely to have financial stability. That much is true.

Commenter 82
I was born in 1950, late in my parents' lives, my father was 47, my mum 45. All my grandparents were born in the 1860s and were dead long before I was born.
My dad died when I was 7, a few months after a serious accident in the colliery where he worked. My mum remained a widow living until she was 93.
As a relatively young parent and grandparent myself I'd recommend you have your children young, they stand the risk of missing so much, as do those who have gone before.
Sadly, nothing in life is that predictable.

Commenter 83
External commenter
"The people in their 70's today aren't like my parents' generation, they are playing golf, going to the gym, travelling and enjoying life, well at least here in Sweden they are."

The bottom 10% by income in the UK would be very unlikely to reach 70. Life expectancy of a woman in the top 5% is 98, that of a woman in the bottom 10% is more than 30 years less.

As for older parents my mother was 38 when I was born and my father 70. My opinion is strictly personal but to have a child at that age is so wrong words fail me.

Commenter 84
I'm 67 and well remember my dad saying he never expected to see me grow up, he was 44 when I was born. Well both parents saw me grow up, get married and have a daughter. Dad died at 86 and mum at the great age of 103. None of us knows how long we have but don't let age put you off kids. I'm privileged to have 2 grandkids which gives me the excuse to be 'naughty'.

Commenter 85
So sympathetic to 'Anonymous' as my experience was similar. My mother was 41 and my father 53 when I was born and I basically gave up my 30s to care for them.
If I thought it was a struggle to maintain a home, relationship, two kids and a decent full time job, but it became impossible when I became the person who drove them to hospital appointments, did their shopping and got called in the night when one or other had fallen, felt unwell or wet the bed. My father died, my relationship of 20 years broke down and I gave up a modest career for a part-time job for the next four years until my mother followed him.
I'm so glad I had my own family in my 20s now, I'd hate to think they had to make the difficult choices I felt pressured into making.

Commenter 86
I am in my early forties. Later this year, I'll be the same age my mom was when I was born, and next year I'll be the age my dad was at that time. He died when I was 19 and she when I was 35. The hardest part has been not having peers experiencing loss and having aging parents at the same time, being around people my age who get to be in some respect parented, to have the feeling of someone looking out for them, even while they raise kids of their own. I think that feeling of being alone in the world has very much contributed to why the idea of having a kid scared me. My mom was never very stable and when my dad went, so did the last dependable adult in my family. (That's an understatement. She was mentally ill and made a lot of messes when she was able to.) My mom and I had a good relationship once I became more responsible for her than age for me, but even then none of my peers were doing anything remotely like this. They had more emotional security and were starting their own families. Obviously there's more to that than the age of my parents but it's really a big part. It's also frustrating that people rarely understand that caring for parents and losing them is as much of a passage into adulthood and responsibly as having kids. Sometimes it feels like friends who have kids but are still so protected emotionally and otherwise by having living parents (and grandparents! I had one and he died when I was eight!) get recognition as adults while I am treated like someone who hasn't grown up and had to take on responsibilities beyond myself because I have no kids and my partner and I are unmarried. Now that many of my age-mates do have aging and dying parents, I've found that I've had to limit the amount of support I give friends on that issue. At first it was a relief no longer to feel like I'm among very few of my friends to know these experiences, but I've had to recognize that now that I've gone through that process, passed that rubicon, and have taken up the mantle of being the oldest generation in my family (of those I have grown up with and have relationships of trust or fealty, all are gone), holding friends' hands though it every day on social media is just a way of reliving an old trauma. I have once or twice gotten very angry when friends my age seen to take lightly the stress and weight I feel of being responsible entirely, emotionally and psychologically, and also financially for everything in my life. I want to say, site you pay your bills like I do, but if you lost your job, you have two living parents with resources, an older sibling with resources, a married partner with those same resources, and grandparents and aunts and uncles to boot who would all take you in. (My dad was the youngest in his family and my mom, bring mentally ill, was not clear to hers.) That's as much

class in some respects but it's also the security of having a family safety net to care for you and advise you, whether in material ways or in a sense of safety in the world, of knowing there's someone you can turn to or sometime you'd better not piss off. In a way I'm jealous of a generation of kids growing up not alone in this. I wish them well.

Commenter 78
Commenter 86
Hear, hear. As the only child of an older mother, who is now in end stage dementia and has been dependent on me for many years (my dad died when I was a student), I do envy my contemporaries, whose parents are still mobile, socially active, cognisant, and emotionally present in their lives.

The early loss of that emotional (and practical) support should not be underestimated; and caring for parents arguably takes more of a toll than caring for children, who grow, develop, and become independent, whereas elderly parents do the reverse. All these things are much easier to accommodate if you have ongoing support yourself, but not all of us do.

Whenever this subject of older parents comes up, focus tends to be on the youth of the child (will I look old at the school gates, will I embarrass my kids at sports day or be unable to kick a ball around the park?) - that's superficial. It's when the child is entering early adulthood and the parent is retiring and becoming frail that the gap becomes an issue - and increasingly so, as the parent becomes more dependent.

Commenter 72
Commenter 86
I know what you mean! I'm 30; I wish I had seen a thread like this when I was 13 and scared out of my skull by Dad's behaviour, my mum's cluelessness, and my little sister's needs, or when I was in my early 20s and felt like a total failure because I hadn't achieved the degree or early-career milestones that my contemporaries had. It's an impossible task, and one that if you speak out about it you risk being branded an ungrateful, unloving wretch. But we're not crazy and we're not alone :)

Commenter 86
Commenter 78
I also arguably experienced career setbacks caring for my mother at a young age, for neither my employer nor the law recognized that I needed time away to do this, and feeling so out of step with my peers I felt it was abnormal, not to be spoken of, and never asked for time away, but eventually my work suffered because I wasn't able to put in extra hours. Indeed, people who were able to get ahead were being supported into adulthood while they did unpaid internships or similar things, not supporting aging parents and putting to rights a lifetime's messy affairs. The support my peers received in graduate school and beyond, while invisible to me, was in fact presumed to be the norm by both my peers and by the institutional structure. While many friends do "get it" there are those who seem to think I'm a desth-obsessed downer, a big stress case, or that I've failed to produce or accomplish what they have. Bedelia is right. Time put into raising children gives you something that grows, becomes independent, and even walks around in the world with you showing others hire you've sent your time. Caring for an increasingly dependent family member is very much not like that.

Commenter 87
My dad was 44 when I was born. I'm 44 now and he's still going strong! My mum is 84. They're both in good health and still happily living in the family home. My best friend had young parents

and were from a much more privileged background than mine, but her dad died before he reached 60. There are no guarantees in life - I love my old dad xxx

Commenter 88

And there are teenagers who father's die from heart attacks at 40, or mother's from cancer at 38.

I'm 34, and both my parents were 41 when I was born. My Dad died aged 75 last year, and I've moved in with my Mam (who has heart failure). The relationship I have with her now is one of the most rewarding I've experienced. I make sure she goes to her hospital appointments, we go out on trips (shopping or to her favourite gardens). I want to make sure we have some quality time together no matter how long we have left together. I've got great plans for Mother's Day, as well as making a big fuss when my sister graduates from Durham University in July.

Ironically, if my parents had been younger when I was born, I wouldn't have worked out that we have a family history of aortic problems. My Gran died in '83 from an aortic aneurysm. My Dad died from an aortic dissection (a tear in the wall of the aorta). I put 2+2 together, and went to the doctor. My sisters and I have since all have had ECGs (I also had a CT as something was picked up in the aortic arch). Had this happened when we where 20 years older, irreversible damage may have already occurred.

Commenter 89

I quite agree with those interviewed here - I was the last child of older parents (my siblings are 13 and 14 years older than I am) and my father died many years ago at what was then nearly regular life expectancy for those of his generation, 72. But I cannot remember I time when I didn't think about and fear losing my parents. I envy my friends who have parents and even grandparents alive. Whatever those who have children later in life may think about looking or feeling "younger" than their years, you can believe me that their children are aware from a very young age that they will be losing out on time with the love and security of their parents. I knew I would watch them all die before me.

Commenter 90

Much as I wish he was still alive, I feel privileged that although I'm only 41 my father served throughout WW2. It makes that war feel more recent and relevant than it does to the average 41 year old. I'm not being presumptuous there; my friends are my age and it was their grandparents who served, not their dads. It's very different. Knowing what he did in his late teens and early 20s keeps me grounded, time and again. I work in digital media for Christ's sake. If I'm a bit stressed about a deadline, the thought of my father heading from Burma to Normandy in a ship that looks like it's been cobbled together like a Blue Peter project on acid usually brings me back to reality.

Commenter 91

I think it hit home that I had a much older dad when our English teacher asked who had a dad over 50 when they were born (we were reading To Kill a Mockingbird).

My dad was 52, my mum a very young 24. They came from China/HK. He was incredibly active, playing tennis, jogging etc. He would tut at our laziness. He went travelling constantly, in his early 80s he was taking trips to Asia, renting apartments for months at a time. I've no doubt this lifestyle would have continued if he hadn't suffered a stroke 10 years ago.

He's still here at 95. He likes to take himself off to Walthamstow Market,or TK Maxx is a fave. But he is much frailer. But it is fascinating listening to his stories of growing up in 1920s China, warlords, keeping red pandas as pets, working in Shanghai after WW2 etc.

Commenter 92
Having older parents is crap. My father was 47 when I was born and 50 when my little sister was born.

As a child you view all adults as old, it was when the teenage years hit, their age becomes another thing to build a barrier, usual teenage angst - but I know my dad feelings were hurt when I basically called him an old man, which as a teenager, he was already in his 60's.

He died when I was only in my 30's and found very little people who could even relate to losing a parent in my circle of friends.

I don't regret my dad, I loved him very much, but god I wished he was around longer.

I'm in my 40's now and looks like I'm doing exactly as he did, (having kids 'late') at least my children will probably have more friends going through the same thing when I go and they haven't even reach 40 yet, as older fathers become more common..

I think that's the hardest thing for children of 'old' parents - they have to say goodbye just as they are grown up enough to have a grown up friendship with them and all the bullshit of the teens, university and being a young hip 20 something are over....

Commenter 93
My father was 44 when I was born, two years younger than I am now. He'd be 92 later this year. Sadly, he was killed by a drunk driver when he was two weeks shy of his 70th.

Having an older father opened up a perspective to me I wouldn't have had otherwise. He fought in WW2, he gave me a brother and a sister old enough to be my own parents, he helped me, unwittingly, understand that "generations" differ greatly and little all at the same time.

What I wouldn't give to be able to care for him today. There was no negative impact on me whatsoever and a whole lot of positive ones.

I miss you dearly and love you with all my heart, Harve.

Commenter 94
My dad was 47 when I was born, my mum 42, and he found himself a widower 3 years later. I can't say that growing up with an ageing parent affected me - ok so he didn't run round like a younger man might have done - but then that's all I ever knew so why would I think it odd? However we had plenty of time to do dad/daughter stuff - my knowledge of the war years is only second hand and I am an ace card player. I've been pretty much self-sufficient all my life as he also worked full time and to me that's a parent's role, to give your children a steady home life and set them on their own path.

Commenter 95
I was born in 1951, my dad was 65 and my mum 35, in those less enlightened days caused a stir in a small market town, especially as dad,s previous marriage had been dissolved through non-consummation. Childhood, despite gossip, family difficulties etc. was wonderful. Late parenting seems to run in the family e.g. my great great grandfather was born in 1813! As for myself I am now the same age as my father when I was born and the proud kinship carer of one of my granddaughters, having had with my wife parental responsibility since she was 9 days old. My main regret being that due to work I was not able to spend as much time with my own children.

Commenter 96

External commenter

"Mine was. Born in 1770 as Captain Cook was landing in Botany Bay!"

I can do even better. My great-great grandfather on my father's side was born in 1757! My great-grandfather was born in 1811, my grandfather in 1855 and my father in 1890. I was born in 1945.

It's very interesting reading people's very different accounts of their experiences.My father married at 50 and was 55 when I was born at the end of WW2. My mother was 35. I had friends with mothers of a similar age - women who had perhaps deferred having children until the end of the war was in sight, but my father was a lot older and that affected me, so I was interested to see posts from people who say they weren't aware that their fathers were older. How wonderful. I remember being very upset because mine, with his 'gammy knee', couldn't run in the Daddy's race at a picnic. Aged 7 or 8, I really upset my mother by wishing on a chicken wishbone that "Daddy was younger". "Don't ever let him hear that!" she said. In my teens I became obsessed with the possibility of him dying in the night as he approached 70.

He worked until he was 72 because we needed the money, and my mother had to endure the humiliation of a colleague of his visiting and asking her to try and persuade him to retire as he wasn't pulling his weight. He was a bit of a hypochondriac but lived to the age of 86, mentally sound but physically frail, whereas my mother died 18 months before him at the age of 66 from a heart attack.

I married at 30, late by standards of those days - an 'elderly primagravida' when my first child was born when I was 33 - even more 'elderly' at 39 when my second was born. I still wish circumstances had enabled me to have children younger. I have always regretted that my children never knew my parents, and I hated the moment when a child in my daughter's class at primary school asked me "Are you Louise's granny?" However my daughter, now 32, says she didn't even realise that I was older until other people in her class said how old their mums were, and it didn't bother her In the least.

I retired at 61, with a state pension and small work pension, earlier than I intended but exhausted by teaching. Will future generations of women, who will have to work until the age of 69/70 to get a state pension, have the energy to deal with teenagers? Of course younger parents can also have health problems or die young, 50 is supposed to be the new 40 (or is it 30?), and today's economic situation means families sometimes need to be started later, but I believe women should think very carefully before deciding to defer their first child until they are over 40. For their own sake and their children's.

Commenter 97

My father was 65 when I was born and my mother 32. Unfortunately my father passed away when I was 14 but I would not have had it any other way. Now almost fourty my wife's (same age as me) had two grandparents pass just this last year and I never even knew any of mine. Her parents are still younger than my father was when I was born which I have kind of just realised.

It's been tough, all of my friends parents are mostly still around and they have enjoyed having a father that saw them graduate school and uni, taught them how to drive, had their first beer with and met their now wives and children. However, the time when my father was alive was amazing although he was incredibly ill for the last few years which made it tough on all of us I always felt he was very aware that he may not be around. As a child I had no idea this was unusual or that I had an older parent than anyone else.

My mother never remarried and of course him passing early had a big impact on me but then again there are plenty of others like me who had a parent die while they were young whether due to accidents or illness, one thing it taught me is that this very much is life.

Commenter 98
My dad was 41 when they had me. Doesn't sound so bad nowadays but in the 80's he didn't want me or my sister to feel we had an old dad so he spent the next twenty years shaving 5 years off his age. My mum knew of course, she even threw him a big 50th birthday party, all the while knowing that he was 55 haha!

He's 75 this month and I am SO close to him. My eight year old thinks his Grandad is magic and they have the most wonderful relationship. Since he's retired, my dad has learned to build a PC & just recently has taken up the Xbox - he & my son play each other at Fifa.

He's had his health problems and I can't bear to think of our lives without him.

Comments to newspaper articles in The Daily Mail Online

Commenter 99
children whose parents look like grandparents, children who will lose those parents at a very early age, children that when they have children there won't be any grandparents | My father was 46 and 49 when I and my sister were born; my mother, 26 and 29. As a child he was mistaken once or twice for my grandfather - yet as my mother was the primary breadwinner my father, throughout his late fifties, managed to take care of two small active girls every single day. He's 65 now and looks nowhere near it due to his active lifestyle. I'm very aware that he's 20 years older than most of my friends' parents. Yet for all this nobody ever seems to have questioned my dad's choice to have kids with the woman he loved, despite the age gap. Yet if it was the other way on, and my mother the older parent, she would be expected presumably to forgo having children because it's "nature's way". Why? Age is what you make it - it has nothing to do with your ability to raise a child.

Commenter 100
my dad was in his late 40's when I was born and I know I missed out on a lot of things my friends where doing with thier younger parents. It was not easy and then he passed away when I was just in my early 20's. I loved my dad dearly but he was already old when I was born. In a way we both missed out on a lot.

Commenter 101
"Not to mention the risk of complications, down syndrome (1 in 5 chance over the age of 40) ! That risk is too great". – External commenter. The risk of Down's Syndrome is 1 in 100 at age 40 but goes up to 1 in 60 at age 42 years old definately not 1 in 5 as you stated. My parents were older, in their 40's when I was born and I was always uncomfortable when I saw them next to the other parents. One of my friends once asked "who's the old woman" when she first saw my mother and I was mortified. I loved my parents but they did not have the ability to deal with a teenager when they were in their 50's and they died when my children were very small.My children don't remember their grandparents which is very sad. Nature has a system for eliminating older women from becoming mothers and Mother Nature knows what she's doing.

Commenter 102
My parents were 42 and 45 when they had me (I'm the youngest of 4). It was hard having older parents as most of my peers had parents who were much younger. I also had a very strict

upbringing and didn't have the freedoms of my friends. Becoming a parent not long before the menopause kicked in also meant my mother took her moods out on me and dumped her "issues" on me. Some of the latter have followed me all of my life and I am now 50. In my late teens my mum died and in my early 40s I was juggling my life with caring for my elderly father. From experience I don't think having older parents is ideal, even though people are healthier and living longer.

Commenter 103

My father was 40 when he started having children (I was the first, followed by two more), and he's the best dad in existence, hands down. I realize 40 is not the same as mid-50s, but I am saying that being a bit older than the standard parent does not make you a worse parent. He's also in fantastic health and the longevity on that side of the family is amazing. I believe he will see my own children graduate high school, and probably even college. (I'm 28 and childless so far... no desire to have kids before 30). Starting a family later in life is definitely not for everyone, but for some people it's perfectly fine.

Commenter 104

My parents were older when I was born. Both knew they had terrible family genetics, ie: heart problems, diabetes, dementia etc. in their family tree. My Father's congenitial health problems started when I was 8 and he was totally disabled through my teenage years and died when I was 21. My Mother started having serious health issues right after his death. At the age of 29, I became my Mother's full time health care giver dealing with heart attacks, strokes, complications of diabetes and finally into mind destroying dementia. All the time, I was holding down a full time job and raising my son, who was born when I was 24. She finally died when I was 41 and left my health in a precarious condition from the years of stress, not to mention it destroyed my marriage, due to the constant care she needed which my husband resented very much for so many years. So, in my opinion, think about a child having to sacrifice their lives to become your full time nurse in case something goes wrong

Commenter 105

My parents had me at the age of 47 years old. They were wonderful and loving but i spent most of my childhood catering to their physical needs only to have them die when I was in my 20's. I am not complaining but please do consider all aspects when having a baby late in life.

Commenter 102

As a product of older parents, I can tell you it can be hard on the kids. My mother, who was 40 when she had me, was not fit to carry another child. She had serious health problems my whole early childhood. This caused bonding issues, and to this day I have intimacy issues and shy away from any affection, because as a young child didn't get it. Not to mention the risk of complications, down syndrome (1 in 5 chance over the age of 40) ! That risk is too great. By the way... mom now has dymentia. I'm in my 30's raising my teenagers which is difficult enough. We've had to move mom in with us because she can't live alone. It's only going to get worse. Often, I think perhaps she should have ignored her selfishness and not had another child so late.

Commenter 106

I'm 30 and my partner, 37, is very keen to have kids. I don't feel as ready as he does but am acutely aware that the later we leave it, the younger our children might be when they lose their dad. My dad was 40 when they had me (youngest of 3) and I'm actually jealous that my older siblings will have had more of my dad than I will. I worry that if we don't have children soon, by the time we do, my dad might not be around or as active as he's been able to be with their

children. So whilst I might not be fully ready myself, I'd rather try to give our children a younger and more active grandfather and dad!

Commenter 107

I'm sick to death of seeing comments about how terrible or impossible it is, for older women to have babies, as there has always been some who do! As a child of an older mother who didn't get married until 40 (no, she would not have been a single mother!) & then after that, went on to have her family, without any form of artificial help, she was just Mum to us & we never noticed any difference in her from other mothers, so to suggest it's bad for the children is nonsense! She also lived long enough to see her grandchildren, let alone her children grow up! We had no physical or mental handicaps (altho' my husband might disagree! LoL!) it entirely depends on the individual woman, as to whether she can handle pregnancy when older. Unlike earlier times, women are NOT "old" at 45 today, so it's very possible for a woman of that age now to cope much more easily with a small child, & maybe a woman who has maturity & patience might be better than a neglectful youngster!

Commenter 108

Just because it's now physically possible doesn't mean it's a good idea. These ladies need to look to the future. I'm an only child aged 42 with an elderly parent (83). My mum didn't choose to have me relatively late (for that time) but now I find myself in a position where I'm looking after two kids, a husband and my Mum (oh and a full time job). Luckily she's reasonably self sufficient for the time being but whilst these older parents may be full of beans now, that won't necessarily be the case in 20 years time.

Commenter 109

I think this is wrong. I remember being teased at school because my dad was old (42 when I was born). But teasing aside, I just don't think it's fair on the children. OK, they'll live long enough to see their children into adulthood, but not very far. If their kids have kids at 30, they will be worrying for aged parents at the same time as small children, and their grandchildren won't have much time with nan and grandad. If they dream of living in a different place, or travelling in their 20s, will they be able to do that without guilt and worry when their 70something parents are at home? Financial security is not the most important thing in raising children. I think it's selfish to put your desire to have children above the emotional wellbeing of those children (and the adults they become. To lose a parent at 30 would be devestating, especially when that's the age people usually start to truly appreciate their parents).

Commenter 110

With the exception of Mrs Palin, every other case here conceived using donor eggs. Egg donation is not something that sits terribly comfortably with me. As a mother (of two, soon to be 3), I could not imagine relinquishing any of my eggs (or babies, to put it another way) to another woman. If I've read correctly, all of the women who struggled to conceive before resorting to donor eggs only began trying for a baby in their late 30s, at the earliest. Women are designed to procreate much earlier than this - and it is safer and healthier to do so. As I've said, I'm expecting my third child - and I am just 29. Becoming a parent at a 'younger' age was something that was important to me, since my father was 48 when I was born and, while he's great and we're still lucky enough to have him around, I wish he were 10 or 20 years younger now! I am not disputing that any of these children will be loved and provided for, but I know I want to be around to be an active grandparent as well as parent.

Commenter 111

Both my parents parents were older when they had me, their only child, I loved them dearly but my life was with my father and now is with my mother spent caring for them and growing up

worrying whether your dad will die from the next heart attack or bout of ill health. I am now full time carer for my mum who is 88. I never wanted that for my children and purposefully had them young.

Commenter 112
My mother was 44 when she had me in the fifties, my dad was 46. My eldest brother was 21 and there were two other siblings in between. My dad died when I was 21 so he never got to walk me down the aisle, as I'd always hoped. Mum died when I was 28. My children never knew their grandparents and I was always envious of other young mum's like me who had their parent's support. My in laws lived 200 miles away, so they weren't around either. Of course I had some family but I've always missed having my parents around for most of my adult life. So on a personal view I think it's selfish to have children later in life.

Commenter 113
My parents were nearly that old when I was born. I always felt very lucky: they were so thrilled to be parents. They'd sowed all their wild oats traveling and partying and were grateful to be home with me. Never wanted time off. They both lived well into their 90s, and I was happy to help them when they got old. We were all very close. I did not wait that long to have my own children, but I definitely know you can be a happy, good parent if that is what life hands you.

Commenter 114
My parents were older when they had me and now I'm having to worry about their failing health and take care of them when I should be enjoying youth and having fun. If you love kids so much and want to feel useful then volunteer your time to a children's charity instead of burdening your own young children down the road.

Commenter 114
Exactly. Its selfish. When they reach their 20s they start having to worry about their parents failing health a nd take care of them when they want to be free and independent.

Commenter 115
I was a surprise when my mom was 47. My youngest sister is 20 years older than me. My mom was a fantastic person & did the best she could- but she absolutely didn't have the energy with me that she did with my siblings. Both my parents began having health issues when i was around 12. I used to pay every night to not lose my mum until I was 25(that just sounds mature to a kid!). I lost her when I was 26, after years of bad health. I barely got to know my oldest brother who was getting his PhD when I was born & them raised his family in another part of the country. I hated people mistaking my parents for my grandparents, it was embarrassing. Having me also decimated their retirement account. I'm happy to be alive, I was definitely loved, but there is no denying things would have been much better had I been born even a decade earlier.

Commenter 116
I had older parents and looking back and through my eyes as a young child I wanted a young, modern trendy and up to date mum like the other children, they were set in their very old fashioned ways and I ended up at 18 years old having to give any hope of uni and nurse them both . I only remember them as old !

Commenter 117
Selfish. Simply selfish. Being an older parent because you chose to postpone motherhood until after nature advised you too means you are tired and the poor child will have to bury you sooner just when they need you. Take it from me, it's not something I'd wish upon anyone

Commenter 118
My mother was 45 and I disagree. Whilst they were good and loving parents they were so out of touch, my teenage years were a nightmare.

Commenter 119
Having a shallow small mined parent would be worse. My parents were old when they had me and I've never once been embarrassed by them, proud of them yes.

Commenter 120
More families will get into the same situation as me and it's hard work. My parents were late 30's when they had me and for the past 3 years I have been my 80 year old dad's main carer. I'm early 40's with a teenage son and every day I have to try be everything to everyone as well as run a business and without an amazing hubby I think I would crack up. I love them all but I do get frustrated by the situation. You expect to be in your mid 50's before you have to think about caring for parents where as I have had it from late 30's. Growing up my parents never had the energy for sporty things and I can't swim or ride a bike. I had my son in my late 20's as I was lucky to marry young and I would urge people to think babies if the situation allows as young as possible. My son too missed out on active grandparents as ill health was setting in as he was a baby so he has no fond memories of days out ect only illness and wheelchairs which is sad

Commenter 121
I loved my parents but as I got older I felt embarrassed as they were in their 40's when they adopted me ,I rarely wanted them to come to parents nights at school as all the other mums were young ,in fact several times I was asked if my mum was my grandmother ,pretty embarrassing for a 12 year old ,I swore never to have a child late in life ,I loved them but sooner stay in doors with them .even on holiday they wanted to sit on a bench and watch the sea pretty boring in your early teens

Commenter 122
I don't think they are selfish as my parents were older, and it didn't harm me..in fact they had money to afford me, and pay for my university, because they were well established and financially able.

Commenter 123
My mum was 40 when she had me nearly 29 years ago. She is not selfish. I am proud of everything she achieved before having children (and after, of course!) If you're fit, healthy and can conceive naturally then whenever YOU and your other half are ready is the right age.

Commenter 124
I was a late in life baby, and I have no complaints. Being in your sixties with teenagers is a rather scary thing. Even a good child can be a terror as a teenager.

Commenter 125
Not selfish, but self-aware, to know when (or not) to have children. As a child of older parents, I had a wonderful childhood. They were solid in their careers, had plenty of time for me and were able to share their life experiences instead of still longing to fulfill them. And young parents or old parents, a child who grows up knowing they are truly loved and wanted has the best start in life.
