# Supplementary material for: An invisible caregiver for visibly older parents: Experiences of (young) adults shared as comments to newspaper articles on advanced age parenthood
Source: PLoS One. 2023 Nov 30;18(11):e0295018. doi: 10.1371/journal.pone.0295018 (PMC10688717; doi:10.1371/journal.pone.0295018)
Supplement: Attachment — Submitted filename: Response to Reviewers.docx [file pone.0295018.s002.docx]

**Response to Reviewers**

Dear Editor,

Dear reviewers,

Thank you for this opportunity to improve our manuscript ‘An invisible caregiver for visibly older parents: Experiences of (young) adults shared as comments to newspaper articles on advanced age parenthood’ and to make it ready for publication in PLOS ONE. We think the reviewers brought up some excellent points which we have tried to address as good as possible. Below, we reply to each point the reviewers raised and explained the requested adjustments we have made.

We hope to have elucidated and sufficiently elaborated all points that were unclear and we are open to additional suggestions to further improve the manuscript.

Sincerely,

Kato Verghote

| **Reviewers’ comments** | **Response** |
| --- | --- |
| **Reviewer 1** | |
| The first condition to increase the probability of being published is to be written, in full, by someone who is native or who has a good expertise of the English language. | We made language changes in all of the sentences the reviewer identified. Moreover, we have asked someone whose mother tongue is English to read through the manuscript and correct additional language issues. Finally, some sentences in the manuscript were removed in order to make the text more understandable and compact. |
| As you refer in the paper, there is a lack of consensus regarding what is considered late parenthood. However, it is important to present what has been considered as such, that is, to present age references, both for the first child and for subsequent ones – What has been considered late parenthood in terms of age, in the literature? | We added a sentence in the introduction to specify what age cut-offs have been used in the current literature on late parenthood: For mothers we found this to range from +30 to +40, and for fathers from +40 to +50. |
| At the end of the introduction, you refer several times to “popular press” – it is important to clarify what popular press is. | We agree ‘popular press’ might have been an inadequate word choice here. Therefore, we changed it to a more specific description of the online spaces where such discussions can take place: “social media platforms, internet forums, and comment sections on other types of websites (e.g., blogs, online newspaper articles)”. |
| At the end of the introduction, you also refer “By studying data stemming from stakeholders expressing in an unsolicited way what they consider relevant for the debate on AAPd we hope to give voice to these stakeholders and initiate further research” (lines 90-92) – Here, I suggest that you analyze whether the “participants” are really stakeholders. Stakeholders are key informants, and, in this case, it seems that they are simply people who volunteer to answer and not people intentionally selected to give their opinion on a concrete subject, in which they have expertise and knowledge.  Also in connection to the word ‘stakeholders’ as used in the methodology section: It is necessary to clarify who are considered stakeholders in this study and what were the criteria for being considered as such. | We interpreted the word ‘stakeholder’ as someone with a relevant experience for the study at hand. Considering the importance of their experience for the literature (given the fact that this is an understudied topic) we see them as key informants, even though we did not intentionally select them as the reviewer correctly indicates. The main criterium for being considered a stakeholder within this research project was that the commenter drew from their own experience with AAPs as they participated in the article’s discussion section.  We tried to clarify this issue at several places in the manuscript by focussing on the importance of the commenters’ personal experience. |
| How did it become clear that the commentator was a child of elderly parents? What were the criteria for being considered as such? | We selected comments in which the commenter either provided general information about their parents’ age (without mentioning the age at birth or the age differences between them and their parents) like “my parents were older when I was born”, or “I had older parents”, or the commenters explicitly stated the age of their parents at the time of the commenter’s birth, which ranged from 38 to 69 years old. The latter was the case for the majority of the comments. These self-defined age references comply with what has been framed in the literature as ‘older parenthood’. We have added a sentence about this in the methods section. |
| Also, one more language issue: it is not correct to say “defined definition”. | Indeed, we changed this to: “Rather than precisely defining ‘older parenthood’…” |
| It is necessary to clarify the meaning of this statement because it is not clear: “The unit of our analysis was comments; therefore, we only made claims about comments and not about the commenters”. | In the methods section we added a sentence to clarify this: “The unit of our analysis consists of newspaper comments rather than the individuals behind the comments (given that the study design was not aimed at gaining an in-depth insight into these individuals). Therefore, in the results below, we make statements about comments and not about the commenters.” |
| In data analysis procedures, you should not name the authors of the article for each task performed. | In the data analysis section, we removed the initials of the authors in relation to each task they performed. |
| It is necessary to clarify the meaning of this statement: “Additional auditing cycles were organised in collaboration with all co-authors”. | We rephrased this as follows: “Auditing meetings were organised in collaboration with all three co-authors. The auditors’ role was to critically challenge and discuss provisional theme structures and to help refine potential themes.” |
| I suggest that, for a better orientation of the reader, you refer right from the start, in each theme, which were the categories (and sub-categories) defined. | We added a couple of sentences to the introduction of each theme, explicitly mentioning the subthemes that will follow in that particular theme. |
| It is necessary to clarify the meaning and purpose of this statement: “Most of the comments formulated by individuals who shared their personal experiences as children of advanced age parents (AAPs) were formulated as both a reaction to the respective newspaper article and a contribution to the debate on advanced age parenthood (AAPd)”. | We decided to remove this part from the manuscript, as the reviewer’s comment made us reconsider the relevance of this sentence. It seems logical that comments to a newspaper article are formulated as a response to the contents of that article. |
| “Overall, there was a comparable number of comments taking a positive and a negative stance towards AAPd.” – What do you mean by comparable? How did you come to that conclusion? Did you count? In what way? | To familiarise ourselves with the collected data during the early stage of analysis, we looked for positive and negative wording in the comments. We counted the comments using ‘more positive wording’ (N = 74) and those using ‘’more negative wording’ (N = 77). In this way, we found “a comparable number of comments using more positive and more negative wording in describing their experiences with AAPd”. However, as this study is qualitative in nature, we do not consider this categorization as part of our main research findings but rather as an introduction to the dataset. |
| When themes are presented (between lines 175 and 181), it is important to put the name of the theme, between parentheses or hyphens. | This was added as suggested. |
| Between lines 210 and 218 several ideas/contents are presented that are not categorized. It is important to create categories/sub-categories for them. These contents are too different from the “Not missing out” category to be integrated into it. | We agree with the reviewer that there are several descriptions between lines 210 and 218 that do not entirely fit the subtheme of “Not missing out”. However, as this concerns only one paragraph, we decided not to create another subtheme. Instead, we removed those descriptions that are presenting something else (e.g., the older parents’ cultural preferences), and kept those descriptions that do relate to the subtheme of “Not missing out” but rephrased them as to make the connection to the subtheme clearer. |
| I suggest that you also create categories/sub-categories within the second and third themes - Being a Child of Visibly Older Parents and Doing the Caring in an Invisible Sort of Way - as they present a lot of rich and different information that can be grouped together and better understood if categories are created to organize it. | With regard to the second theme we added 2 subcategories in order to enhance understanding: (i) Sensing difference, (ii) Dealing with social judgement.  With regard to the third theme we added 3 subcategories in order to enhance understanding: (i) Timing of caregiving, (ii) Societal ignorance, (iii) Emotional costs of caregiving.  We also added a short paragraph at the beginning of each theme in which we introduce the subcategories that will be presented in that specific theme. |
| **Reviewer 2** | |
| In the Introduction section (lines 80-82) the authors stated: "The only available qualitative accounts of (adult) children born to older parents date from the last century and are more journalistic in nature..." Since these are the only qualitative studies regarding (adult) children born to older parents, a deep analysis of these studies' results is needed. | A summary of these studies’ main findings was added to the introduction. |
| Also in the Introduction section, the authors should indicate more clearly what are the main contributions to the literature of this research. | In the penultimate paragraph of the introduction we added a sentence to clarify the main contributions of our research: “With this study we aim to provide an updated explorative analysis of the experiences of (young) adults born to AAPs and to foster societal understanding of what it means to these people to be a caregiver for their parents at a relatively young age.” |
| In the Method section, the authors should indicate why they used reflexive inductive thematic analysis and the benefits of using this approach. | In the section ‘Data analysis’ we have now elaborated on our aim to use thematic analysis, as well as on the benefits of using this approach. |
| Besides, since the audience of the analyzed newspapers is global, meaning readers are from several countries, the authors should state this. | We added a statement about this in the section ‘Study Site’. |
| In the Discussion section, the authors focused mainly on the third theme: the invisibility of taking up an untimely caregiver role for AAPs. An in-depth discussion of the remaining two themes would be necessary to highlight the study's results correctly. | We added some discussion of our first two themes at the beginning of the discussion section. Moreover, we tried to outline why the largest part of the discussion section relates to the third theme of our results. |
| Regarding the third theme, the authors did not present/discuss the main differences between young adult children caregivers and older adult children caregivers regarding burdensome caregiver responsibilities that adversely affect their lives. Are these two groups so different? | In the first version of the manuscript we have submitted, we tried to outline the main similarities and differences between underage and young adult caregivers, but not between young adult and older adult caregivers. This is because it is not always possible to distinguish between these groups. It was difficult to make strict categories like ‘young adults’ and ‘older adults’ in our dataset for a variety of reasons: 1) comments were often limited in the sense that they lacked background information (like: for how many years the commenter had been a caregiver, when the caregiving had started, or when it had stopped), 2) life stages like ‘young adulthood’ and ‘older adulthood’ could be interpreted in different ways by the commenters (one may call oneself a ‘young adult’ at the age of 30, whereas another may not, for example), and 3) some commenters described caring for their parents for over more than a decade, therefore possibly having been a caregiver as a young adult *and* an older adult. Also, we are not certain whether there were many ‘older adult’ caregivers in the comments as burdensome caregiving experiences were mainly described during one’s young adult life. For these reasons we decided not to make a distinction between young adult and older adult caregivers in the results section. If deemed necessary by the reviewers/Editor, we could include this commentary in the manuscript. |
| The authors should highlight the study's practical implications and state some public social and health policies to support these younger caregivers. | We have added a paragraph near the end of the manuscript in which we discuss the need for an increased sensitivity of social policy to anticipate younger and older individuals’ informal care activities alike. Moreover, we pointed to the need for the education system and social services to identify young caregivers and offer them support and a safe space to talk openly about their caregiving experiences. |
